# A Prospective Randomized Study to Predict Bowel Preparation Quality Prior to Colonoscopy: Comparison of Two Stool Collection Methods for the Objective Assessment of Final Rectal Effluent Clarity

**DOI:** 10.3390/diagnostics15131717

**Published:** 2025-07-05

**Authors:** Serdar Senol, Mustafa Kusak, Kevser Uzunoglu Yıldırım, Mustafa Gun, Mıne Gızem Bıdıl

**Affiliations:** 1 Department of Surgical Gastroenterology, Samsun University, Ilkadım 55090, Samsun, Türkiye; 2 Department of General Surgery, Samsun Training and Research Hospital, Ilkadım 55090, Samsun, Türkiye; mkusak75@yahoo.com; 3 Department of Surgical Gastroenterology, Samsun Training and Research Hospital, Ilkadım 55090, Samsun, Türkiye; dr.kevseruzunoglu@gmail.com; 4 Department of General Surgery, Samsun University, Ilkadım 55090, Samsun, Türkiye; dr.mstfgn@gmail.com (M.G.); minegizembidil@gmail.com (M.G.B.)

**Keywords:** bowel, clarity, colon, perception, preparation, sensitivity, stool

## Abstract

**Background/Objectives**: Adequate bowel preparation is essential for high-quality colonoscopy. The clarity of the final rectal effluent can predict its sufficiency and guide additional preparation if necessary. For an objective and reliable clarity assessment, the stool collection method may be as important as the evaluation itself. This study was designed to compare the sensitivity of clarity assessments of effluent collected using two methods: a disposable cardboard bedpan with a white bag (Group I) and a 50 mL transparent plastic container (Group II). **Methods**: A prospective, single-center, randomized, comparative study was conducted between August 2024 and January 2025. Based on predefined criteria, 37 of 177 randomized patients were excluded, and 140 were analyzed. **Results**: Inadequate bowel preparation was correctly identified by a team member in 71% of Group I and 23% of Group II (*p* = 0.033). In adequate cases, the detection sensitivity was 88% and 85% (Groups I and II, respectively; *p* = 0.854). Significantly more patients in Group II either withdrew or failed to submit a photograph of the final rectal effluent. Patients’ verbal assessments did not differ significantly between the groups, regardless of bowel preparation quality. **Conclusions**: Patient self-assessment was an unreliable indicator of bowel cleanliness, highlighting the need for objective, standardized pre-colonoscopy evaluation methods. The use of a disposable cardboard bedpan with a white bag to collect the final rectal effluent may improve the accuracy of predicting inadequate preparation and patient compliance and may allow timely adjustments to bowel cleansing prior to colonoscopy in routine endoscopy practice.

## 1. Introduction

Colorectal cancer is one of the leading causes of cancer-related deaths in the world. Its incidence and mortality rates are declining due to the development of screening methods [1,2], of which colonoscopy is the most sensitive and is recommended by the most recent guidelines [3,4].

The adequacy of bowel preparation, the colonoscopy withdrawal time, appropriate polypectomy, complications of the procedure, the cecal intubation rate, and the adenoma detection rate are described as colonoscopy quality indicators [5]. Bowel preparation has important impacts on the other factors and may represent the basis of high-quality colonoscopy [6]. Furthermore, inadequate bowel preparation decreases the cecal intubation rate and follow-up intervals and increases the procedure time and complication rate [7,8,9,10]. It is also correlated with a lower adenoma detection rate, which may result in the development of interval colorectal cancers [11,12,13,14]. The American Gastroenterological Association (AGA) recommends achieving adequate bowel preparation in ≥90% of screening colonoscopies [14].

Many patient-related variables, such as higher body mass index, older age, men with diabetes mellitus, stroke, dementia, and antidepressants use, have been identified as risk factors, and inadequate bowel preparation has been reported in up to 20% of colonoscopies [15]. Additionally, the quality of bowel preparation is closely associated with patient adherence to written and verbal instructions regarding the details of bowel preparation and dietary restrictions, which are defined as standard patient instructions. These requirements are often complex and difficult for patients to comprehend. Outpatients can easily forget key details of the instructions during a long waiting time between the delivery of instructions and the scheduled colonoscopy [16]. Enhanced patient instructions, such as telephone calls, videos, and social media applications, are used as reminders to improve patients’ adherence [17]. In recent years, mobile phones have become increasingly popular in every age group, and telephone calls may be the preferred method of receiving instructions, as suggested in a recent meta-analysis that included nine randomized controlled trials [18].

Several scales are used to assess the quality of bowel preparation. The Boston Bowel Preparation Scale is one of the most validated assessment tools [19,20,21,22]. A score of ≥2 in each colon segment corresponds with adequate bowel preparation. Unfortunately, the quality of bowel preparation can be assessed only after the endoscopist has inserted the colonoscope. The European Society of Gastrointestinal Endoscopy recommends repeated examination within one year if colonoscopy is performed with inadequate bowel preparation [7,23]. However, with this strategy, a significant proportion of these patients may be missed [24,25], and all steps of the procedure need to be rescheduled for a future session.

A description of the clarity of the final rectal effluent may be used to predict the actual bowel preparation status just prior to colonoscopy. This evaluation may lead to a change in management by administering more laxatives or enemas before proceeding with colonoscopy [7,26]. Although the literature on this topic spans approximately two decades, few researchers have focused on the subject, and evidence is limited. The initial articles were based on patient self-assessment of the final rectal effluent and reported that patients’ perceptions poorly correlated with actual bowel cleansing [27,28]. Subsequently, photographic examples of rectal effluent were used to improve the correlation between patients’ perceptions and endoscopists’ assessments [29]. However, photographic-example-guided patient descriptions of the last rectal effluent had low clinical significance. As emphasized in a recent prospective study, both the last rectal effluent examination and the methods used to collect effluent are critical to the objective assessment and prediction of actual bowel preparation [30]. Nevertheless, the results and limitations of this study were the subject of a new study. Collecting a sample of rectal effluent in a 50 mL plastic container is not easy, and non-compliance with a warning not to mix the collected sample with flush water is a potential problem for the examination. Carrying a plastic container filled with a stool sample may also cause reluctance and prevent deterioration. We hypothesized that guidance based on the clarity of the last rectal effluent directly collected from the anal output in a disposable cardboard bedpan is easier and may make the assessment more accurate by increasing patient compliance with warnings. Additionally, having patients or someone else take photographs rather than bring the stool sample in a plastic container may decrease their reluctance and deterioration. Therefore, a prospective, single-center, randomized, comparative study was planned in order to compare the sensitivity of the objectively assessed clarity of the last rectal effluent collected using two methods: direct collection from the anal output in a disposable cardboard bedpan with a white bag and in a 50 mL plastic container. Additionally, we aimed to determine whether the method used to collect the last rectal sample affected patients’ adherence to the research instructions. The study also incorporated another secondary objective: the sensitivity of patients’ verbal descriptions of their last rectal effluent’s clarity.

## 2. Materials and Methods

Study design: In the endoscopic center of a surgical gastroenterology unit, a prospective, single-center, randomized study was conducted to compare the sensitivity of the objectively assessed clarity of the last rectal effluent collected using two methods: direct collection from the anal output in a disposable cardboard bedpan with a white bag and in a 50 mL plastic container.

Ethics and informed consent statement: The study was approved by the Ethics Committee (2024/13/6). Written informed consent was obtained from all enrolled patients. The trial conformed to the ethical guidelines of the Declaration of Helsinki and Good Clinical Practice.

Patient population and randomization: Potential participants were screened when they visited the surgical gastroenterology outpatient clinic. Those who were 18 years of age or older, had no history of colorectal resection, and agreed to provide written informed consent were considered eligible for the study. They were randomized to directly collect the last rectal effluent in a disposable cardboard bedpan with a white bag or a 50-mL transparent plastic container. Randomization was conducted by using sequentially numbered and sealed envelopes, prepared by a team member blinded to the endoscopy procedures. Even numbers were assigned to the disposable cardboard bedpan group (Group I), and odd numbers were assigned to the 50 mL transparent container group (Group II).

**Figure 1:**
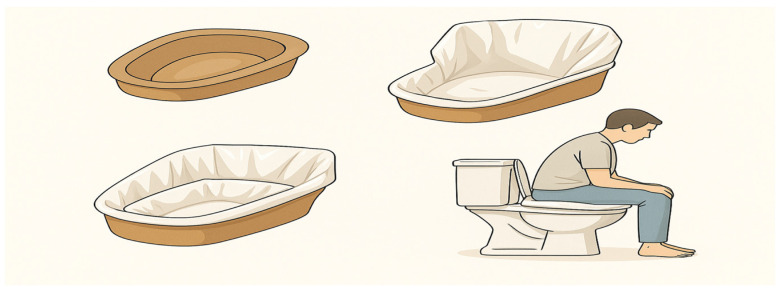
Practical application of disposable cardboard bedpan with white bag.

Setup and practical application of disposable cardboard bedpan with white bag in clinical settings: Each patient was given a disposable cardboard bedpan with a white bag to collect the last rectal contents. In addition, three white plastic bags were available for reuse if necessary. Each patient was instructed on how to use the bag and disposable cardboard bedpan. The base of the bag was placed on the inner surface of the cardboard bedpan. The edges of the plastic bag were folded under the bedpan to ensure that the inner surface was completely covered by the bag (Figure 1). Patients were advised to position themselves over the prepared bedpan to defecate. For those with access to a commode-type toilet at home, it was recommended that they place the bedpan directly onto the chair before defecating.

Investigation of the impact of the methods used to collect the final stool sample on patient compliance: Some previous studies have highlighted that withdrawal of consent and failure to provide requested photographs may be quantifiable and objective indicators of patient compliance with a study protocol [31,32,33]. Therefore, the distribution of participants who either withdrew from the study or failed to provide photographs was analyzed in both groups, with the goal of determining the effect of the method used to collect the final rectal effluent on patient adherence.

Bowel preparation: All patients received a standard paper-based explanation in addition to telephone-based enhanced education about diet restrictions and purgative instructions. Two days before the procedure, patients were contacted via SMS, and the instruction to maintain a liquid diet was re-emphasized. One day before the colonoscopy, patients were contacted via telephone, and instructions on bowel preparation were re-emphasized. Patients received two 250 mL bottles of senna solution, each containing 500 mg of sennoside a + b. On the day of the colonoscopy, participants were requested to take a photograph of the last rectal effluent with a phone camera with at least a 10-megapixel resolution capacity and asked to send it as a phone message via an application. The image was captured prior to the colonoscopy procedure, after the completion of all bowel preparation steps and the final bowel movement.

Bowel preparation adequacy criteria: Bowel preparation adequacy was assessed using the Boston Bowel Preparation Scale (BBPS), a validated instrument that scores each of the three colonic segments—right, transverse, and left—on a scale from 0 to 3 (0: poor; 1: fair; 2: good; 3: excellent; Table 1), with a total score range from 0 to 9. A preparation was considered adequate if the total BBPS score was ≥6 and no individual segment scored <2. Any score not meeting these criteria was defined as inadequate [19].

**Figure 2:**
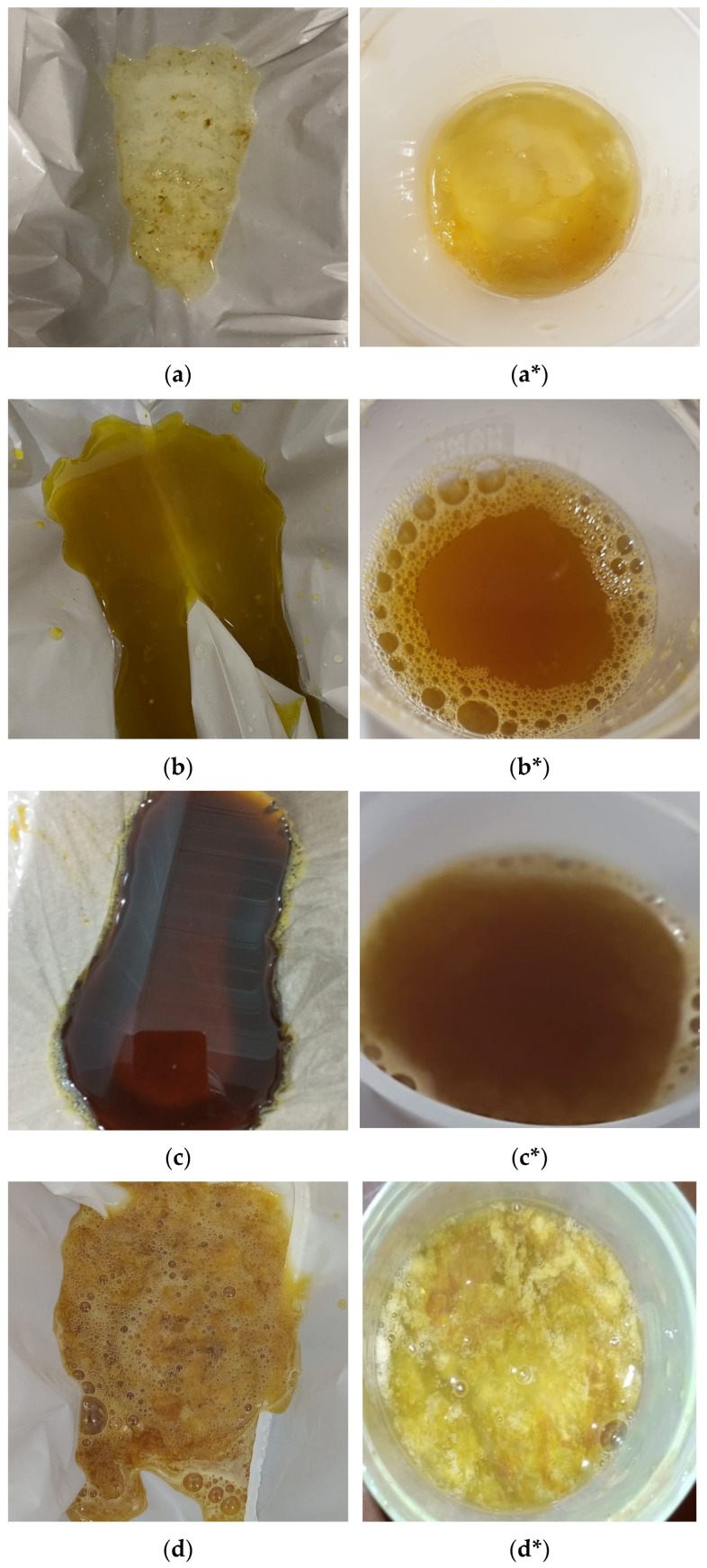

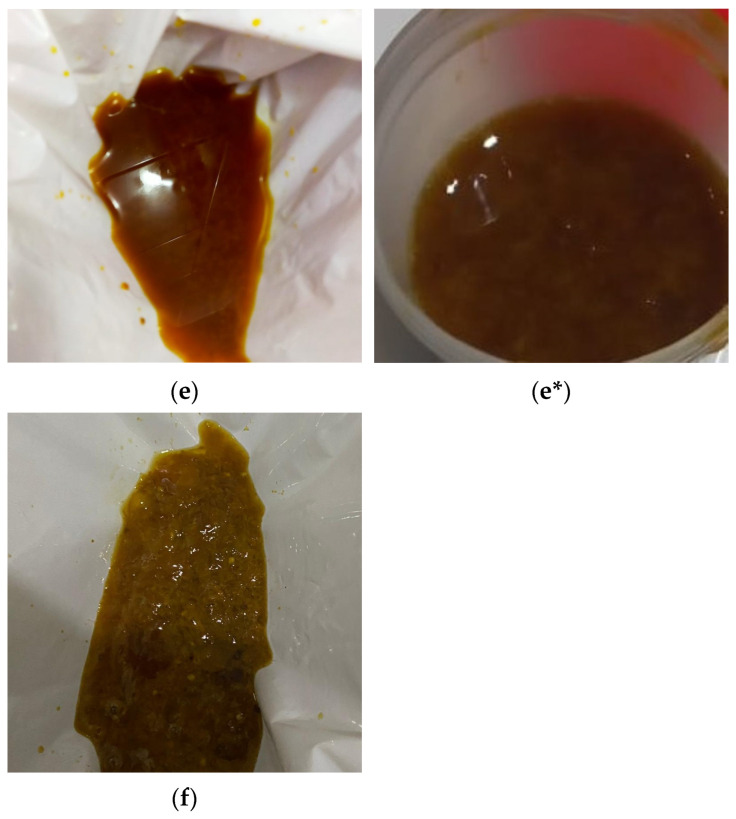
The clarity scale of the last rectal effluent. The clarity of the last rectal effluent was categorized in order of increasing density and decreasing clarity as follows: (**a**,a*) thin clear fluid; (**b**,b*) thin yellow fluid; (**c**,c*) thin brown fluid; (**d**,d*) thick yellow fluid; (**e**,e*) thick brown fluid; (**f**) particulate matter; (*) was used to define whether the last rectal effluent was directly collected in a disposable cardboard bedpan with a white bag or a 50 mL transparent plastic container, respectively.

Assessment of outcomes: The clarity of the last rectal effluent in the photographs was categorized by a team member blinded to the endoscopy procedures, in accordance with a recent study [30], and illustrated using clarity codes, as shown in Figure 2.

Another team member used a questionnaire (Appendix A) to record the consumption of the bowel preparation agent, the total number of bowel movements after the last dose of senna solution, the time interval between the last defecation and the beginning of the colonoscopy, and the patient’s verbal description of the last rectal effluent using stool clarity codes (see Appendix A, last question). All of the colonoscopy procedures were performed in the morning session. High-resolution video-colonoscopy (Fujinon Corp., VP-7000 processor, EC-760R-V/L colonoscope, Fujifilm Corporation, Tokyo, Japan) was used for the assessment. All manipulations of the colonoscope were performed by a single endoscopist who was blinded to the interpretation of the last rectal effluent photographs and the results of the questionnaire. Procedural factors were recorded, including, bowel preparation quality, cecal intubation and extubation times, and the findings of the colonoscopy. Bowel preparation quality was classified as adequate (total score ≥6 with no segmental score <2) or inadequate (total score <6 or any segment <2) based on the BBPS.

Exclusion criteria included (1) patients who withdrew from the study; (2) patients who did not take a photograph of the last rectal effluent on the day of the colonoscopy; (3) patients who did not adhere to dietary restrictions and/or purgative instructions; (4) patients with failed cecal intubation.

Sample size: The sample size for the study was calculated by utilizing the G*Power software (version 3.1.9.7). It was determined that the minimum sample size was 62 participants with an effect size of 0.85, an error (α) of 0.05, and a power (1-β) of 90%.

Statistical analysis: Descriptive statistics were calculated to provide information about the general characteristics of the working groups. Data for quantitative variables were defined using mean and standard deviation (x ± ss); data for qualitative variables were defined using number (*n*) and percentage (%). Normality assessment for numerical variables was performed using the Kolmogorov–Smirnov test. Continuous variables were compared between Group I and II, using the Independent Samples *t*-test (Student’s *t*-test), while categorical ones were analyzed using Pearson and Fisher’s exact Chi-square. Significance analyses were performed to investigate the effect of the method used to collect the final stool sample on patient compliance. The sensitivity for each group was analyzed to assess the accuracy of both team members’ evaluations and patients’ verbal descriptions of the last rectal effluent, and the Chi-square tests were utilized to determine the statistical significance of the differences between Groups I and II. The statistical significance was set to a *p*-value of less than 0.05. All calculations were performed using SPSS 22 (IBM Corporation. 2013. IBM SPSS Statistics for Windows, version 22.0. Somers, NY, USA: IBM Corporation).

## 3. Results

Between August 2024 and January 2025, 177 patients were identified as eligible for the study. Eligible patients were 18 years or older, had no history of colorectal resection, and agreed to provide written informed consent. They were randomized to collect the last rectal effluent directly in a disposable cardboard bedpan with a white bag or in a 50 mL transparent plastic container. Randomization was conducted using sequentially numbered and sealed envelopes, with even numbers being assigned to the disposable cardboard bedpan group (Group I) and odd numbers assigned to the 50 mL transparent container group (Group II). There were 88 patients in Group I and 89 patients in Group II.

Failed cecal intubation occurred in three patients, two of whom were in the disposable cardboard bedpan group. This was because of tumor obstruction at the rectosigmoid junction and a redundant colon. Nine patients—five in the disposable cardboard bedpan group and four in the transparent container group—withdrew from the study. In the transparent container group, 14 patients did not take a photograph of the last rectal effluent on the day of the colonoscopy. Eleven patients—eight in the disposable cardboard bedpan group and three in the transparent container group—did not follow dietary restrictions and/or purgative instructions. After excluding these patients, a total of 140 patients—73 in the disposable cardboard bedpan group and 67 in the transparent container group—were finally included in the study.

In order to investigate whether the methods used to collect the final stool sample affected patient compliance, significance analyses were performed, which included all patients who withdrew from the study and those who did not take a photograph of the final rectal content. The results revealed that the majority of patients were in Group II, and the difference was statistically significant (*p:* 0.011).

### 3.1. Patient Demographic and Clinical Characteristics

The mean ages in Groups I and II were 50.9 ± 12.6 years and 54.9 ± 12.2 years, respectively (*p*: 0.06). The demographic composition of both groups exhibited a preponderance of female participants, with no statistically significant disparities observed between the groups in relation to gender distribution. More than three-quarters of the patients in each of the two groups had an American Society of Anesthesiologists (ASA) classification score of two or three. Patients with at least one additional disease accounted for 46.6% and 44.8% of Groups I and II, respectively. Hypertension and diabetes were the most frequent co-existing disorders in both groups. The data did not reveal a statistically significant difference between the groups with respect to ASA classification score or comorbidities. Additionally, there were no statistically significant differences in body mass index, academic level, previous abdominal surgery, or prior insufficient bowel preparation between patients in each group. The most common indications for the procedure included screening colonoscopy, constipation, and anemia. The others were rectal blood loss, diarrhea, and surveillance colonoscopy due to a prior polypectomy procedure. The indications were not significantly different between the groups. The details of their data, together with other basic demographic and clinical characteristics of the groups, are shown in Table 2.

### 3.2. Preprocedural and Intraprocedural Characteristics and Colonoscopy Findings

The analysis of the data revealed no statistically significant differences in the mean number of defecations subsequent to the last senna dose (7.61 ± 3.92 vs. 8.10 ± 4.09; *p*: 0.408) or the mean interval between the last defecation and the beginning of the colonoscopy (157.08 ± 99.90 min vs. 176.07 ± 105.18 min; *p*: 0.275). In addition, the mean cecal intubation and extubation times exhibited no statistically significant differences (7.23 ± 2.87 min vs. 6.49 ± 2.37 min; *p*: 0.118; 8.29 ± 3.00 min vs. 7.87 ± 2.37 min; *p*: 0.415, for Groups I and II, respectively). The total procedural time was shorter in Group II (13.2 ± 3.95 min vs. 14.99 ± 4.54 min; *p*: 0.022).

The ratios of adequate bowel preparation were nearly identical in both groups, with 80.8% in Group I and 80.6% in Group II achieving adequate preparation (*p*: 0.999). The number of patients with at least one adenoma was comparable between the two groups (18 in Group I vs. 20 in Group II). Similarly, the total number of detected adenomas was nearly identical, with 31 in Group I and 30 in Group II. When adenoma sizes were analyzed, no significant differences were found between the groups. Both groups had 15 adenomas smaller than 5 mm, while Group I had 16 adenomas equal to or larger than 5 mm, compared to 15 in Group II (*p*: 1.000 and *p*: 0.857, respectively). The adenoma detection rate (ADR) was slightly higher in Group II than in Group I (36.3% vs. 30.5%), although this difference was not statistically significant (*p*: 0.465). Other colonoscopy findings, including diverticula (five in Group I vs. seven in Group II; *p*: 0.564), colon cancer (two vs. one; *p*: 0.564), and inflammatory bowel disease (one vs. three; *p*: 0.317), did not differ significantly between the groups. Anal fissures and parasites were observed only in Group I, but due to their low incidence, no statistical comparison was made.

### 3.3. The Clarity of the Last Rectal Effluent, Sensitivity of Assessment by the Team Member and Patients for Each Group, and Evaluation of Statistical Significance

The clarity of the last rectal effluent in the photographs was labelled by a team member blinded to the endoscopy procedures, in accordance with a recent study [30], and was classified as 1: thin clear liquid; 2: thin yellow liquid; 3: thin brown liquid; 4: thick yellow liquid; 5: thick brown liquid; and 6: particulate matter. Patients’ verbal descriptions of the last rectal effluent were recorded by a team member using a questionnaire with the same clarity codes (Appendix A; last question). The patients in each group were subdivided into subgroups based on actual bowel preparation status: those with adequate bowel cleansing and those with inadequate bowel cleansing. The details are shown in Table 3.

The stool clarity codes for patients in each subgroup were regrouped to include team member responses and patients’ verbal responses. Stool clarity codes 1, 2, and 3 were considered to represent adequate bowel cleansing, while codes 4, 5, and 6 were considered to represent inadequate bowel cleansing. The team member correctly identified 52 of 59 (88%) patients with adequate bowel preparation in Group I, whereas patients’ self-assessment yielded a sensitivity of only 42% (25 out of 59 patients). Similarly, in Group II, the sensitivity of the team’s classification was 85% (46 of 54 patients), while patients’ assessments remained at 37% (20/54). Among patients with inadequate bowel preparation, the team member identified 71% of Group I and 23% of Group II (10 out of 14 and 3 out of 13 patients, respectively), whereas patients correctly identified only 29% (4/14) and 15% (2/13), respectively.

**Figure 3:**
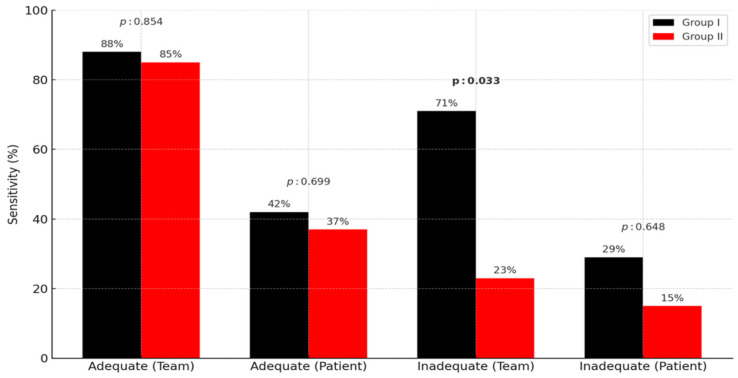
Sensitivity of clarity classification by team and patients in each group.

Although the team member’s classification of patients with inadequate bowel preparation differed significantly between Groups I and II (*p*: 0.033), there was no significant difference in the evaluation of patients with adequate bowel preparation between groups. The verbal assessments of the patients were not significantly different between Groups I and II, regardless of whether patients had adequate bowel cleansing or inadequate bowel cleansing. These results are summarized in Figure 3.

## 4. Discussion

Based on the limited available evidence collected by a few investigators on this topic, both the last rectal effluent assessment and the method used to collect the effluent are critical to making an objective assessment to predict actual bowel preparation [26,27,28,29,30]. In this prospective, single-center, randomized study, we initially compared the sensitivity of the objectively assessed clarity of the last rectal effluent collected using two methods: direct collection from the anal output in a disposable cardboard bedpan with a white bag (Group I) and collection in a 50 mL plastic container (Group II). In predicting patients with adequate bowel preparation, the team members had high and comparable sensitivity in both groups (88% in Group I and 85% in Group II), and there was no statistically significant difference (*p*: 0.854). Conversely, in patients with inadequate preparation, the identification rate was significantly higher in Group I than in Group II (71%, 10/14 vs. 23%, 3/13, respectively; *p*: 0.033). In this context, we may infer that collecting the last intestinal contents in a disposable cardboard bedpan with a white bag increases the ability to accurately predict inadequate bowel cleansing. Furthermore, the superior sensitivity observed in Group I can be attributed to several factors that may collectively improve the prediction. First, the white bag provides a uniform, high-contrast background, which facilitates a clearer visual distinction between effluent colors and consistencies, enabling the detection of subtle differences. Second, the bedpan’s larger surface area allows for more even spreading of the effluent, minimizing pooling and supporting a more comprehensive assessment of clarity and particulate content. Third, collecting effluent directly from the anal output in a disposable cardboard bedpan may help maintain its original properties by avoiding dilution or alteration that could occur during transfer or storage in a small plastic container. These observations are consistent with the recent findings of Patwa et al., who demonstrated that the objective assessment of rectal effluent in a transparent container significantly enhanced the prediction of bowel cleansing quality, while water-diluted samples exhibited reduced reliability [30]. Similarly, So et al. emphasized the importance of background contrast and direct visualization by demonstrating that visual guidance improved assessment accuracy [29]. Therefore, it can be concluded that, in comparison to a patient with adequate bowel cleansing and light-yellow or light-brown clarity codes, a patient with inadequate bowel cleansing and dark-yellow or dark-brown clarity codes is more likely to be influenced by this potential inaccurate assessment.

The question of identifying patients at high risk of inadequate preparation prior to colonoscopy and improving the quality of bowel preparation prior to sedation was first raised in a study conducted by Harewood et al. in 2004, which was designed to assess the accuracy of patients’ perceptions of the quality of bowel preparation [27]. In addition to the findings of Harewood et al., Fatima and colleagues demonstrated that patients’ perceptions were insufficient to predict the actual bowel preparation status prior to the colonoscopy procedure [27,28]. Hoonsub et al. used photographic examples of the last rectal effluent to improve patients’ perceptions. However, patients’ photographic-example-guided perception of the last rectal effluent had low clinical significance [29]. The results of a recent study also support this finding [30]. In accordance with these studies, the perceptions of patients in our study were not sufficient to predict actual bowel preparation, regardless of whether patients had adequate or inadequate bowel cleansing.

According to some studies, taking photographs or videos and withdrawing from a study have been identified as measurable and objective criteria of patient adherence to the research instructions [31,32,33]. In the current investigation, an evaluation of these measures for each group revealed that the number of patients who did not take photographs and those who gave up participation in the study were significantly more represented in Group II. This discrepancy may be attributed to several factors. First, as compared to a disposable cardboard bedpan, the smaller and narrower opening of the plastic container may have created practical difficulties during sample collection. Second, participants may have perceived the method as less hygienic or visually unpleasant, particularly when compared to the use of a disposable cardboard bedpan with a white bag. These findings suggest that the method of collection may directly influence patient adherence in addition to data quality and potential pre-colonoscopy decision-making in clinical settings.

The internal validity of this study is supported by the lack of statistically significant differences in baseline demographic and clinical characteristics between the two groups. Variables such as age, sex, body mass index, comorbidities, ASA classification, and history of previous bowel preparation were well balanced, and this reduced the number of possible confounding influences. Moreover, the groups were comparable in terms of preprocedural and procedural factors, including the number of bowel movements after the last senna dose and the time interval between the last bowel movement and the beginning of the colonoscopy. Adequate bowel preparation is a necessity for high-quality colonoscopy [7,14], and in our study, both groups achieved comparable rates of adequate bowel preparation (80.8% vs. 80.6%; *p*: 0.999). These results are consistent with previous reports, which revealed that insufficient bowel preparation may occur in approximately 18% to 35% of patients undergoing colonoscopy [7,15]; however, these rates slightly exceed the acceptable upper limit of 10–15% recommended by international guidelines [8,14]. This highlights the ongoing challenge of achieving adequate bowel preparation despite efforts, including enhanced patient education, split-dose drug administration, and a shortened time interval between preparation and the beginning of the procedure. In addition, this underscores the clinical value of reliably predicting bowel cleanliness before the procedure to allow timely intervention in daily practices [26]. Interestingly, the total procedure time was the only procedural parameter that differed significantly between groups. Despite the total colonoscopy duration being significantly longer in Group I (14.99 ± 4.54 min vs. 13.2 ± 3.95 min; *p* = 0.022), this discrepancy does not appear to be attributable to procedural complexity or detection performance. The rates of adequate bowel preparation were found to be nearly identical between the two groups (80.8% vs. 80.6%; *p*: 0.999). Similarly, the adenoma detection rates were comparable (30.5% vs. 36.3%; *p*: 0.465). The cecal intubation times (7.23 ± 2.87 vs. 6.49 ± 2.37 min; *p*: 0.118) and extubation times (8.29 ± 3.00 vs. 7.87 ± 2.37 min; *p*: 0.415) also demonstrated no significant difference. Given that the endoscopist was unaware of the effluent’s appearance during the procedures, the observed time difference cannot be attributed to any anticipatory visual bias. A more probable explanation may be attributable to random variation, subtle unmeasured factors such as patient anatomy or tolerance, or procedural pacing. Subsequent studies devised to evaluate intraprocedural behaviors in a real-time setting may offer further insights into this modest but statistically significant difference.

The main limitations of this prospective study include its single-center design and relatively small sample size, which may restrict the generalizability of the findings. While the study was sufficiently powered to assess primary outcomes, a broader patient population across multiple centers would enhance external validity. According to current international guidelines, the minimum acceptable adenoma detection rate (ADR) is ≥30% [14]. Additionally, these guidelines emphasize the importance of monitoring serrated lesion detection rates (SDRs) and recommend that endoscopy units report and receive feedback on SDR performance. However, given the relatively low prevalence of serrated lesions, it is advised that such assessments be based on at least 500 screening colonoscopies to ensure statistical reliability. Although our study did not reach this threshold, which limits the reliability of serrated lesion detection, the ADR results observed in both groups remained consistent with previously reported values, thereby supporting the clinical validity of our main findings. It should also be noted that the present study focused on the objective assessment of last rectal effluent clarity in predicting bowel preparation quality and ADR was not assessed in patients with inadequate bowel preparation because inadequate cleansing prevents reliable mucosal assessment and introduces significant confounding factors. Future studies with larger sample sizes could explore ADR in this subgroup, although its clinical relevance under such circumstances remains limited [12,13]. On the other hand, no statistically significant difference was observed in the comparison of ADR between patients with adequate bowel cleansing. Although the ADR depends on the combined effect of multiple variables [12,13,34], no significant differences were detected between groups in terms of demographic and clinical characteristics, including bowel cleansing, which is the most important determinant for ADR. These findings suggest that the white-bagged disposable bedpan may facilitate a better assessment of preparation quality. However, its use does not appear to have directly influenced ADR in our study. Additionally, while photographic assessments were conducted by a team member blinded to the endoscopic findings, visual characteristics within the photographs unintentionally disclosed the collection method, and this might have introduced a potential source of bias that could not be fully eliminated. Nevertheless, in order to strengthen the objectivity of the evaluation, the evaluator was blinded to the endoscopic procedures and received structured training with a standardized image reference set prior to the study to minimize subjectivity.

Unfortunately, inadequate bowel preparation has remained the most common cause of unsatisfactory colonoscopy and there is no agreement on the ideal management after bowel preparation failure. The European Society of Gastrointestinal Endoscopy and US Multi-Society Task Force (US-MSTF) recommends rescheduling the procedure within one year in cases with inadequate bowel preparation [23,35]. US-MSTF defines alternative salvage methods as follows: administration of a large volume enema for patients reporting brown last rectal effluent; same-day or next-day colonoscopy after ingesting more oral solution; and through-the-scope enema with completion [35]. In clinical practice, this simple and low-cost method could assist endoscopists in deciding whether to proceed with colonoscopy or to initiate additional cleansing measures such as administration of oral laxatives or enemas. Additionally, building on this idea, emerging evidence suggests that smartphone-based imaging combined with artificial intelligence (AI) could standardize this evaluation [36,37,38], and future studies should validate AI models specifically on images captured in white-bagged bedpans and assess clinical outcomes such as ADR, patient compliance, and cost-effectiveness.

## 5. Conclusions

The present study has once again demonstrated that patient self-assessment is an unreliable indicator of bowel cleansing, as evidenced by the substantially lower sensitivity rates compared to the team-based assessments, thus highlighting the necessity for more objective and standardized preprocedural assessment methods. The use of a disposable cardboard bedpan with a white bag to collect the final rectal effluent enabled greater sensitivity in predicting inadequate bowel preparation, indicating its potential value as a more objective tool in preprocedural evaluation and routine endoscopy practice. Moreover, the observed higher overall patient compliance in the disposable cardboard bedpan group suggests that collection method may influence patient adherence. These findings require further investigation to assess whether such methods could facilitate timely and effective interventions in routine endoscopy practice, particularly when integrated with smartphone-based applications supported by artificial intelligence for real-time effluent evaluation.

## Figures and Tables

**Table 1:** Boston Bowel Preparation Scale.

Score	Explanation
0	Visualization of the mucosa was not possible due to the presence of solid stool
1	Liquid and semisolid feces resulted in the visualization of a segment of the mucosa
2	Minimal residual intestinal content was present and did not interfere with the observation of the mucosa
3	No residual intestinal content was present; and the mucosa was clearly observed.

**Table 2:** Baseline demographic and clinical characteristics.

	Group I (*n* = 73)	Group II (*n* = 67)	*p* Value
Age, mean (SD), years	50.9 ± 1.6	54.9 ± 12.2	0.060
Gender, *n* (%)			0.819
Male	33 (45.2)	29 (43.3)	
Female	40 (54.8)	38 (56.7)	
Body mass index, (kg/m^2^), mean (SD)	27.43 ± 4.6	27.9 ± 4.7	0.494
Education level, *n* (%)			0.994
Illiterate, elementary, secondary	37 (50.68)	34 (50.74)	
High school, college	36 (49.32)	33 (49.26)	
Comorbid conditions	34 (46.6)	30 (44.8)	
Hypertension	27	19	0.238
Diabetes mellitus	11	15	0.317
Coronary artery disease	9	7	0.617
Hypothyroidism	4	4	1.000
COPD	2	3	0.655
Depression	1	1	*
Multiple sclerosis	1	0	*
Cerebrovascular accident	1	0	*
ASA classification score, *n* (%)			0.491
ASA 1	14 (19.2)	10 (14.9)	
ASA 2	53 (72.6)	54 (80.6)	
ASA 3	6 (8.2)	3 (4.5)	
Previous abdominal surgery, *n* (%)	25 (34.2)	29 (43.2)	0.586
Previous inadequate bowel preparation, *n* (%)	4 (5.5)	8 (11.9)	0.248
Indication, *n* (%)			0.954
Screening for colon cancer	27 (36.9)	25 (37.4)	
Constipation	22 (30.1)	18 (26.8)	
Anemia	9 (12.4)	8 (11.9)	
Rectal blood loss	8 (10.9)	8 (11.9)	
Diarrhea	4 (5.5)	3 (4.5)	
Surveillance (polypectomy)	3 (4.2)	5 (7.5)	

SD: standard deviation; COPD: chronic obstructive pulmonary disease; ASA classification: American Society of Anesthesiologists’ classification; *: Not calculated due to low frequency.

**Table 3:** (A) The distribution of stool clarity codes in patients with adequate bowel preparation. (B) The distribution of stool clarity codes in patients with inadequate bowel preparation.

**(A)**				
**Clarity of Stool Sample**	**Group I** **Team Member’s Classification**	**Group I Patients’ Verbal** **Description**	**Group II** **Team Member’s Classification**	**Group II** **Patients’ Verbal** **Description**
(1) Thin clear liquid	1	0	3	0
(2) Thin yellow liquid	29	14	22	14
(3) Thin brown liquid	22	21	21	6
(4) Thick yellow liquid	3	15	1	13
(5) Thick brown liquid	4	9	7	21
(6) Particulate matter	0	0	0	0
Total number	59	59	54	54
**(B)**				
**Clarity of Stool Sample**	**Group I** **Team Member’s Classification**	**Group I** **Patients’ Verbal** **Description**	**Group II** **Team Member’s** **Classification**	**Group II** **Patients’ Verbal** **Description**
(1) Thin clear liquid	0	1	2	0
(2) Thin yellow liquid	2	7	3	7
(3) Thin brown liquid	2	2	4	4
(4) Thick yellow liquid	2	1	0	2
(5) Thick brown liquid	5	3	4	0
(6) Particulate matter	3	0	0	0
Total number	14	14	13	13

## Data Availability

The data presented in this study are available on request from the corresponding author due to legal restrictions.

## Appendix A

Have you consumed any solid food in the last three days? Yes/No
Have you been able to adapt to fluid consumption in the last three days? Yes/No
Have you used all of your medication before the procedure? Yes/No
What time was it when you had your last dose of senna?
How many bowel movements did you have after the last dose of senna?
What time was it when you had your last bowel movement?
What colour was your last rectal effluent?
  (1) thin clear fluid
  (2) thin yellow fluid
  (3) thin brown fluid
  (4) thick yellow fluid
  (5) thick brown fluid
  (6) particulate matter
